# Bottom-up effects on herbivore-induced plant defences: a case study based on compositional patterns of rhizosphere microbial communities

**DOI:** 10.1038/s41598-017-06714-x

**Published:** 2017-07-24

**Authors:** Emilio Benítez, Daniel Paredes, Estefanía Rodríguez, Diana Aldana, Mónica González, Rogelio Nogales, Mercedes Campos, Beatriz Moreno

**Affiliations:** 1 Estación Experimental del Zaidín (EEZ), CSIC, 18008 Granada, Spain; 2 Estación Experimental Las Palmerillas, Cajamar, Almería Spain; 3 Instituto de Investigación y Formación Agraria y Pesquera, Centro IFAPA La Mojonera, Almería, Spain

## Abstract

Below-ground soil microorganisms can modulate above-ground plant-insect interactions. It still needs to be determined whether this is a direct effect of single species or an indirect effect of shifts in soil microbial community assemblages. Evaluation of the soil microbiome as a whole is critical for understanding multi-trophic interactions, including those mediated by volatiles involving plants, herbivorous insects, predators/parasitoids and microorganisms. We implemented a regulated system comprising *Nerium oleander* plants grown in soil initially containing a sterile/non sterile inoculum, herbivore *Aphis nerii* and predator *Chrysoperla carnea*. After aphid attack, plants emitted a characteristic blend of volatiles derived from two biosynthetic classes*:* fatty acid catabolites and aromatic-derived products. Three aliphatic compounds were mainly detected in plants grown in the inoculated microbial soil, a blend which was preferentially chosen by *C. carnea* adult females. The contrasting effect of the initial inocula was attributed to the different microbial consortia developed in each treatment. We argue that differences in the relative abundance of the active microbial communities in the rhizosphere correlate with those in the emission of selected volatile compounds by attacked plants. The mechanisms involved in how the functional soil microbiome modulates inducible indirect defence of plants are discussed.

## Introduction

As part of the evolutionary adaptation process, phytophagous insect-plant interactions occur according to the behavioural choices of insects and the development of physical and chemical plant defences^1^. In addition, volatile plant secondary metabolites possibly function as signals in communications with other organisms in the environment. In this context, the plant-mediated effects on predator-prey and host-parasitoid interactions in tri-trophic systems of herbivore-induced plant volatiles (HIPVs) have been well-documented^2^. The release of HIPVs, generally a mixture of green-leaf volatiles, terpenes and aromatic compounds, among others^3^, may signal the presence of potential prey or hosts and therefore can be exploited by natural enemies to locate the prey organism. Pioneering plant studies show how chewers, sap feeders and herbivore egg deposition induce the production of volatiles attractive to entomophagous arthropods^4–7^.

In addition to indirect interactions, plants act as a link between above- and below-ground communities. It is well known that soil-borne non-pathogenic microbes can modulate plant-insect above-ground interactions via plant growth promotion or induced systemic resistance by triggering biochemical changes in the primary plant metabolism^8^. From a multi-trophic perspective, the tri-trophic role of plant secondary chemistry has been shown to be central to an understanding of various aspects of trophic phenomena, including top-down and bottom-up regulation of herbivores. In recent years, several studies have investigated the effects of below-ground microbes on the third trophic level organisms via changes in HIPV emission^9^. However, most studies focusing on modifications in plant volatile emission through interactions with soil microorganisms mainly address plant interactions with single species of non-pathogenic microbes, which have a neutral, synergistic or antagonistic effect on plant secondary chemistry^10, 11^. Given the significant impact of microbial community diversity and richness on plant signalling pathways, the root microbiome as a whole needs to be considered in relation to many aspects of plant immunity^12, 13^ such as induced indirect defence and insect population dynamics. Nevertheless, to date, no overall trend has emerged in relation to the effects of increased microbial complexity on microbe-plant-insect interactions, with some evidence showing a significant, limited or zero impact on above-ground herbivores^14^.

**Figure 1:**
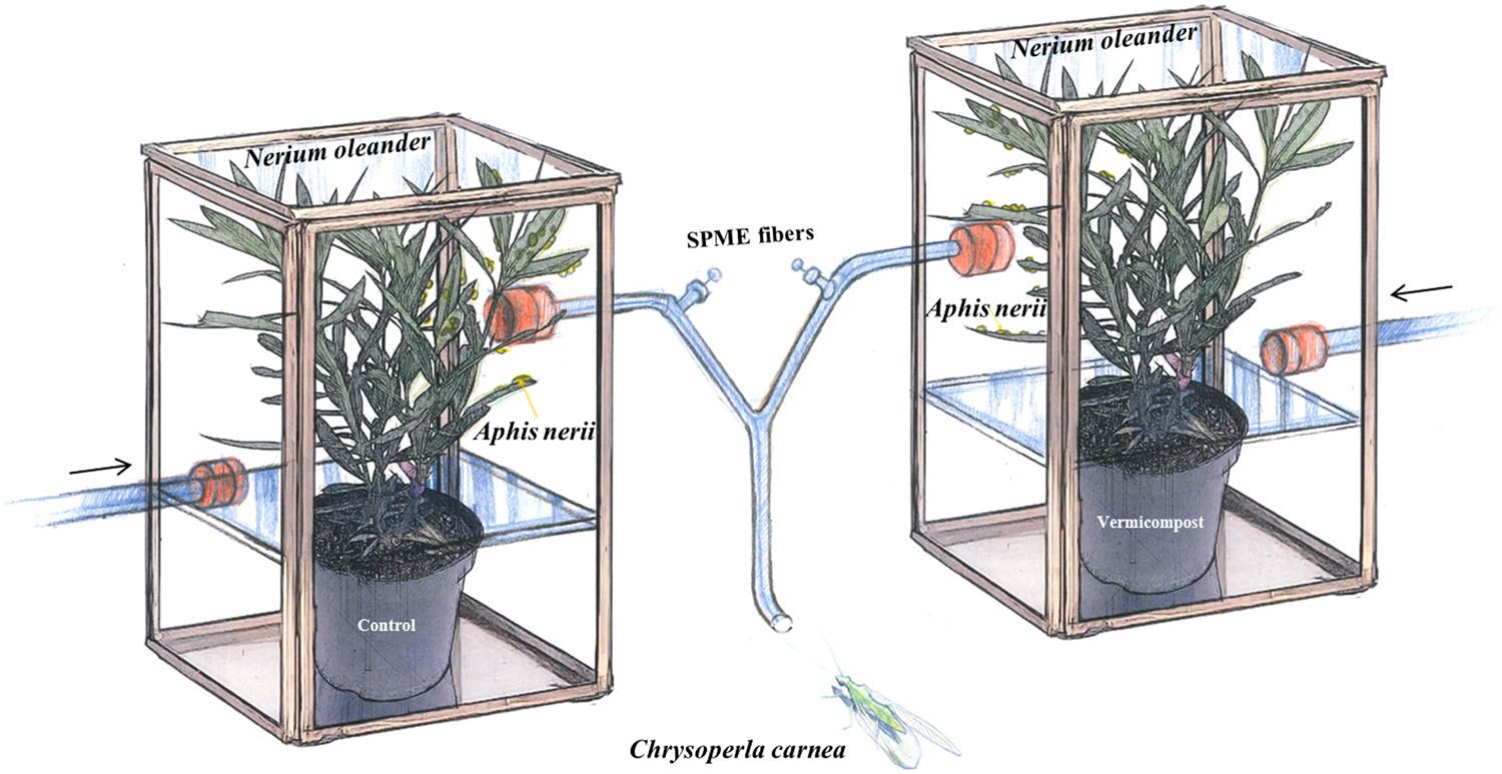
Regulated olfactometer system comprising *Nerium oleander* plants grown in a potting soil initially containing a sterile (Control)/non sterile (Vermicompost) inoculum, the herbivorous *Aphis nerii* and the generalist predator *Chrysoperla carnea*.

In this study, we aim to evaluate the role of the rhizosphere microbiome in HIPV production and its impact on above-ground community interactions. In a case study, we designed a system-based model with three trophic levels, with *Nerium oleander* as host plant, *Aphis nerii* as phloem-sap feeder and *Chrysoperla carnea* as predator (Fig. 1). The plants were grown for 3 months in sterilized soil with a microbial inoculant and were then infested with *A. nerii*. The HIPVs produced were sampled using solid-phase micro-extraction (SPME) and were measured by GC-MS. A Y-tube olfactometer was used to investigate the orientational response of *C. carnea* to the plant volatiles. Using a metagenomic approach, we analyzed the composition and diversity of entire and active microbial communities.

## Results

### *Chrysoperla carnea* behaviour

**Figure 2:**
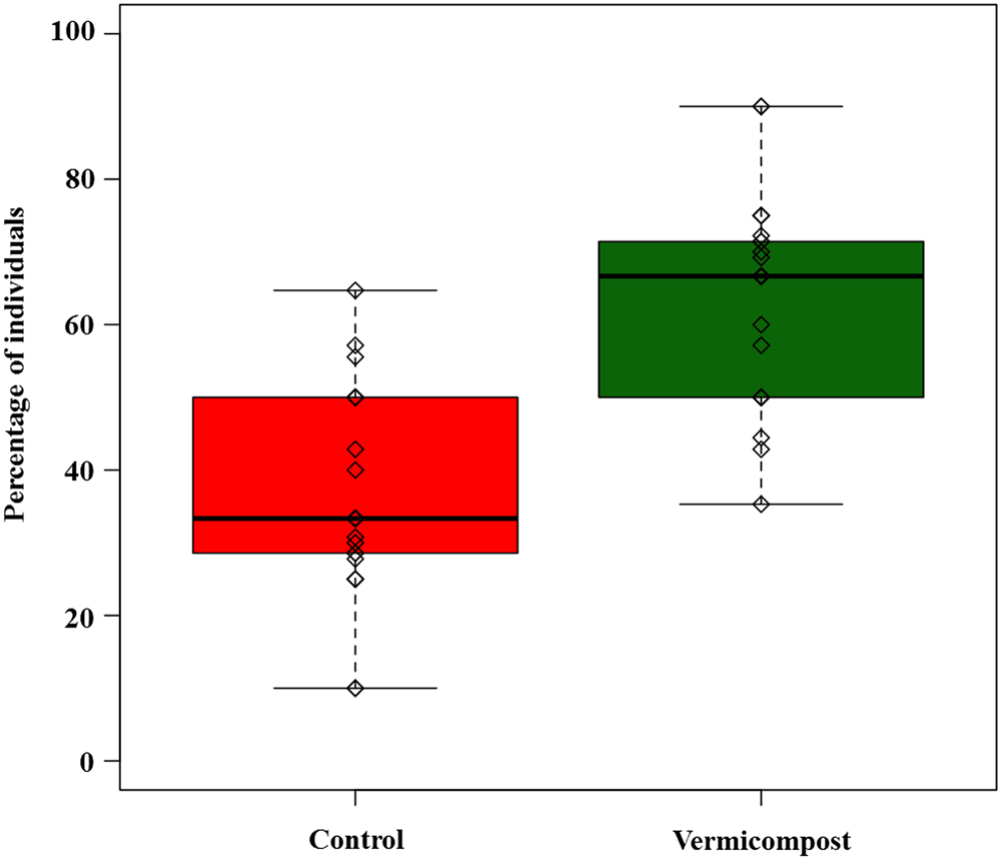
Percentage of *Chrysoperla carnea* moving towards plants of *Nerium oleander* grown in a potting soil initially containing a sterile (Control)/non sterile (Vermicompost) inoculum and after *Aphis nerii* attack.

Model selection indicated that the most parsimonious model only takes account of fixed factor treatment without considering block and assay variables (AICc = 167.21, ΔAICc = 2, R2 = 0.25). Selected model estimated 2.55 *C. carnea* individuals chose the branch connected with the sterilised vermicompost treatment, whereas the non-sterilized vermicompost treatment was chosen by a predicted total average of 4.32 *C. carnea* individuals. This means that 37.12% of individuals chose the Control treatment, while 62.88% selected the Vermicompost treatment (Fig. 2). The results indicate a clear preference of female *C. carnea* adults for the volatile compounds produced under vermicompost soil treatment conditions.

### Volatiles

**Figure 3:**
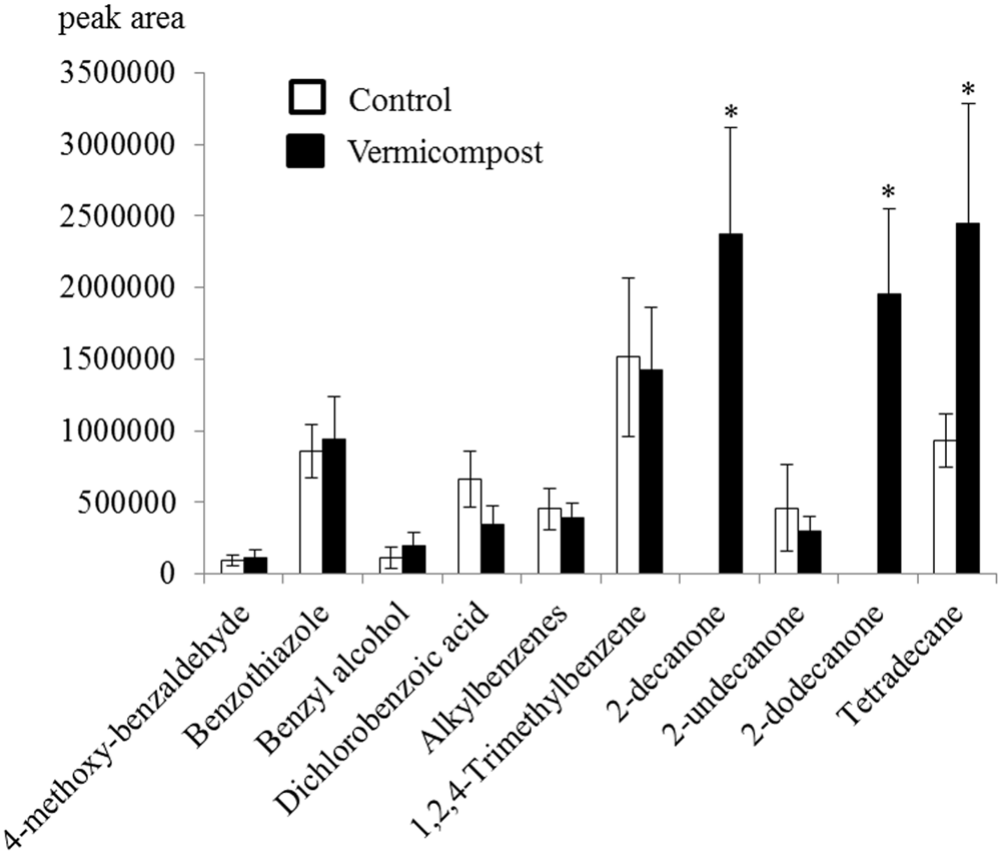
Volatiles (mean ± SE) emitted by *Nerium oleander* plants grown in a potting soil initially containing a sterile (Control)/non sterile (Vermicompost) inoculum and after attack by *Aphis nerii. V*_*1*_: 4-methoxy-benzaldehyde (P = 0.600, F = 0.323), *V*_*2*_: benzothiazole (P = 0.627, F = 0.276), *V*_*3*_: benzyl alcohol (P = 0.091, F = 4.902), *V*_*4*_: dichlorobenzoic acid (P = 0.090, F = 4.962), *V*_*5*_: alkylbenzenes (P = 0.403, F = 0.873), *V*_*6*_: 1,2,4-trimethylbenzene (P = 0.831, F = 0.052), *V*_*7*_: 2-decanone (P = 0.001, F = 62.381), *V*_*8*_: 2-undecanone (P = 0.830, F = 0.053), *V*_*9*_: 2-dodecanone (P = 0.001, F = 63.562), *V*_*10*_: tetradecane (P = 0.005, F = 33.091).

The capacity of *N. oleander* to emit volatiles has been previously described^15, 16^. However, the volatile blend emitted by *N. oleander* plants under non-attack conditions was quantitatively insufficient to be detected by SPME-GC in our experiment. Depending on the potting soil media used, *N. oleander* plants damaged by *A. nerii* under laboratory conditions emitted a different variety of volatile organic compounds 3 days after infestation (Fig. 3). The 2-decanone, 2-dodecanone and tetradecane compounds were mostly detected when plants were grown in the microbial inoculated soil (Vermicompost treatment).

### *Nerium oleander* dry weight and chemical characteristics of soil samples

After harvest, no differences in the dry weight of *N. oleander* plants between the two treatments were detected (Control = 62.91 g, Vermicompost = 64.57 g; F = 0.213, P = 0.656). Detailed information on potting soil characteristics can be found as Supplementary Table S1. As expected, fractional sterilization had no significant effect on the intrinsic chemical properties of the vermicompost^17^. No significant differences were detected between the two treatments, with values for pH, C:N ratio and macro and micronutrients found to be comparable in Control and Vermicompost soils at the end of the experiment.

### Composition of active and whole microbial communities

After 3 months of incubation, the two microbial communities (from a sterile and natural vermicompost) showed different taxonomical structures when inoculated in the same sterile soil. We retrieved over 160,000 bacterial and fungal operational taxonomic units (OTUs) from potting soils previously surveyed by pyrosequencing rRNA gene and gene transcript amplicons.

### Bacterial communities

**Figure 4:**
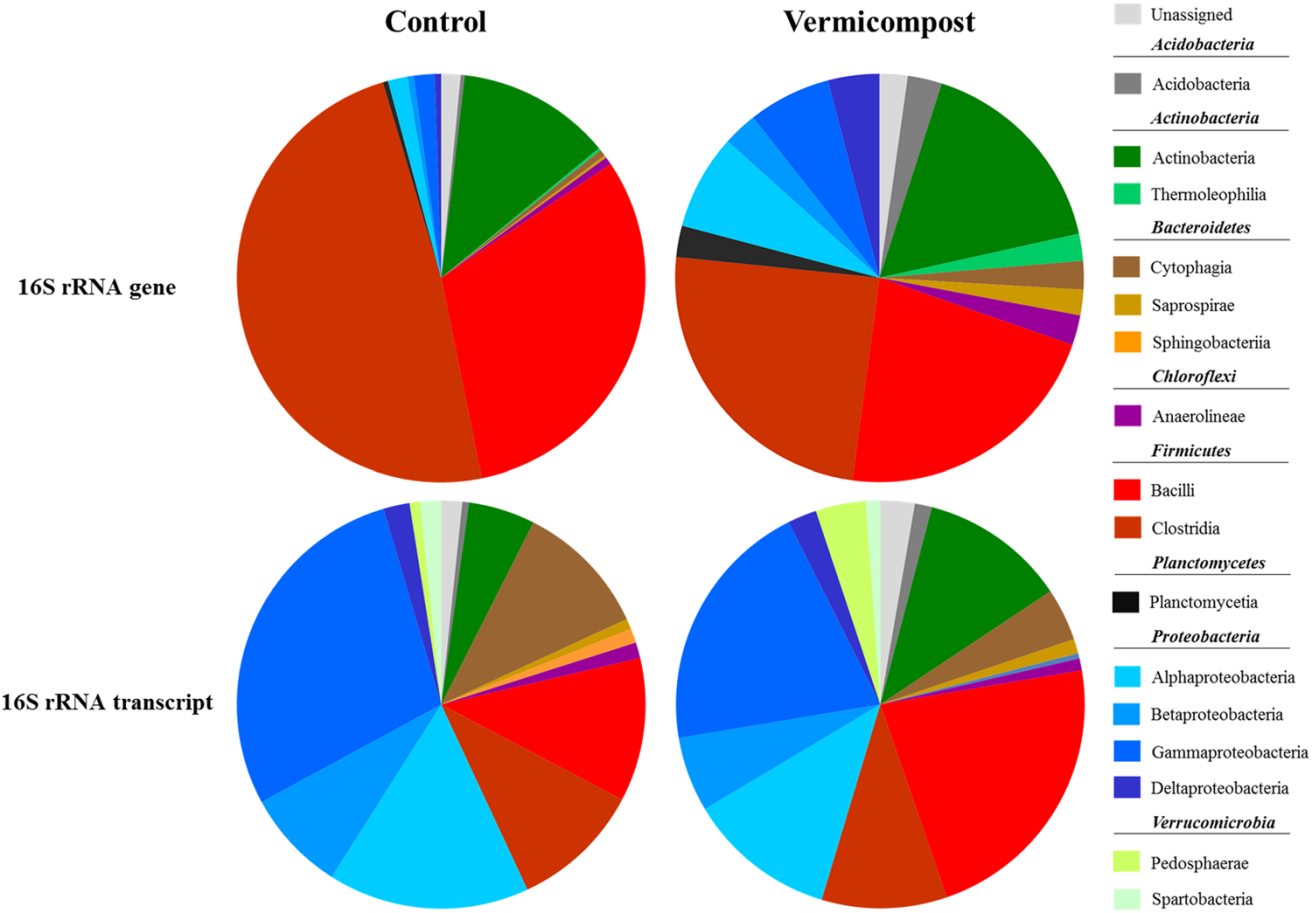
Relative abundance of total (16S rRNA gene) and active (16S rRNA transcript) bacteria in potting soil initially containing a sterile (Control)/non sterile (Vermicompost) inoculum.

Eight dominant phyla (abundance > 1%) were present in the rhizosphere soil samples, accounting for more than 99% of all bacterial sequences (Fig. 4). The total bacterial community was dominated by *Firmicutes* (Control 79.49%, Vermicompost 46.07%), *Actinobacteria* (Control 12.14%, Vermicompost 16.52%) and *Proteobacteria* (Control 4.18%, Vermicompost 20.79%). Its composition differed significantly between treatments, with, most notably, a single phylum, *Firmicutes*, accounting for almost 80% of the entire bacterial population in the Control treatment. On closer analysis, no particular differences between treatments in terms of genus or species were detected, although relative abundance within bacterial classes did vary across treatments (Supplementary Fig. S1). For example, we observed differences between relative abundances of orders *Bifidobacteriales* (97% Control, 54% Vermicompost) and *Actinomycetales* (6% Control, 35% Vermicompost) in *Actinobacteria*, genera *Lactobacillus* (94% Control, 76% Vermicompost) and *Clostridium* (72% Control, 48% Vermicompost) in *Bacilli* and *Clostridia*, respectively, and between relative abundances of orders *Pseudomonadales* (62% Control, 28% Vermicompost) and *Xanthomonadales* (28% Control, 46% Vermicompost) in *Gammaproteobacteria*.

With respect to the active bacterial community, the differences in relative abundance at class level were less marked between treatments than those detected in the total bacterial community (Fig. 4). The phylum *Verrucomicrobia*, which was detected at the DNA level with insufficient relative abundance (<1%), was identified at the RNA level with some differences observed in relative abundance at the order level (Supplementary Figs S2, S3). We also detected differences between relative abundances of orders *Actinomycetales* in *Actinobacteria* and order *Caulobacteriales* in *Alfaproteobacteria* (21% Control, 9% Vermicompost) as examples. The richness indices showed a similar comparative trend in terms of predicting the number of OTUs in all soil samples, displaying those from the Vermicompost treatment the highest alpha diversity -*Chao1* and Faith’s Phylogenetic Diversity- values at both the DNA and RNA levels (Supplementary Fig. S4). Beta diversity patterns in the four datasets (Vermicompost DNA, Control DNA, Vermicompost RNA, Control RNA) were examined using qualitative and quantitative similarity indices at the putative species level (Table 1). The Sorensen-Dice distance and Bray-Curtis dissimilarity indices, based on presence/absence and abundance data, respectively, showed a high degree of differentiation among bacterial communities, particularly between total and active populations in treatment Control. At the RNA level, the Bray-Curtis dissimilarity index also displayed a certain degree of similarity between treatments at the RNA level. UniFrac distances, incorporating information on relative relatedness of community members through the inclusion of the phylogenetic distances between organisms, reflected a similar trend. Both the weighted (quantitative) and unweighted (qualitative) variants of UniFrac point to a medium-to-high degree of dissimilarity in bacterial population structure both in and between treatments at the DNA and RNA levels. It is important to note that the number of denoised sequences (62.160 and 49.730 per sample of DNA and RNA, respectively) is considered an enough sequencing depth to describe a trend in both alpha and beta diversity^18^.

**Table 1:** Beta diversity patterns in the four datasets (VC DNA, C DNA, VC RNA, C RNA) examined using Sörensen-Dice Distance (*SD.D*), Bray–Curtis Dissimilarity (*BC.D*), Unweighted UniFrac (*UW.U*) and Weighted UniFrac (*W.U*) indexes.

		C DNA	C RNA	VC DNA
C RNA	*SD.D*	0.818		
	*BC.D*	0.857		
	*UW.U*	0.763		
	*W.U*	0.581		
VC DNA	*SD.D*	0.753		
	*BC.D*	0.728		
	*UW.U*	0.720		
	*W.U*	0.343		
VC RNA	*SD.D*		0.761	0.793
	*BC.D*		0.567	0.633
	*UW.U*		0.700	0.739
	*W.U*		0.244	0.320

C: soil initially containing a sterile inoculum; VC: soil initially containing a non-sterile inoculum.

### Fungal communities

**Figure 5:**
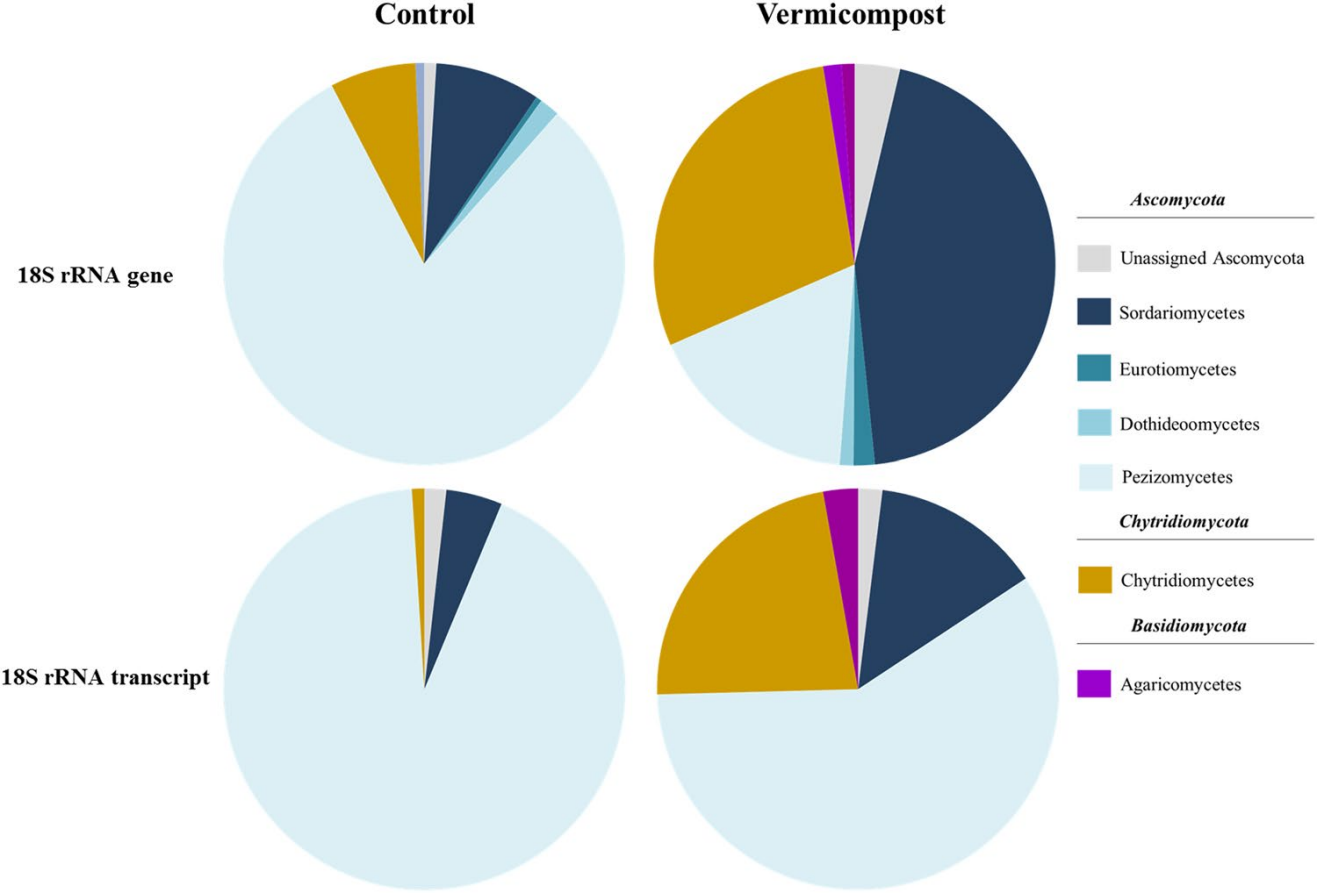
Relative abundance of total (18S rRNA gene) and active (18S rRNA transcript) fungi in potting soil initially containing a sterile (Control)/non sterile (Vermicompost) inoculum.

Three dominant phyla accounting for over 99% of all fungal sequences were present in the rhizosphere soil samples. The number of assigned reads was 152.643, 136.583, 128.412 and 137.465 for Control-DNA, -RNA, and Vermicompost-DNA and -RNA sets, respectively. The total and active fungal communities were dominated by two phyla, *Ascomycota* and *Chytridiomycota*, with remarkable differences between the two treatments (Fig. 5). While neither total nor active communities ascribed to *Ascomycota* changed significantly following each treatment (Control DNA 91.52%, Vermicompost DNA 67.93%; Control RNA 97.75%, Vermicompost RNA 74.07%), *Chytridiomycota* varied to some extent in terms of relative abundance in treatment Control at the RNA level (Control DNA 7.52%, Vermicompost DNA 30.30%; Control RNA not detected, Vermicompost RNA 22.45%). Fungal species belonging to phylum *Basiodiomycota* were only detected at the DNA level. In class terms, the main differences between treatments at the DNA level were found with respect to phylum *Ascomycota*, particularly in classes *Sordaromycetes* (Control 8.37%, Vermicompost 44.39%) and *Pezizomycetes* (Control 80.2%, Vermicompost 17.2%). The latter was also the predominant active fungal class in both treatments, particularly in rhizosphere soil Control (relative abundance 92%). Class *Chytridiomycetes* accounted for all *Chytridiomycota* fungi at the DNA and RNA level.

### Microbial data analysis

**Figure 6:**
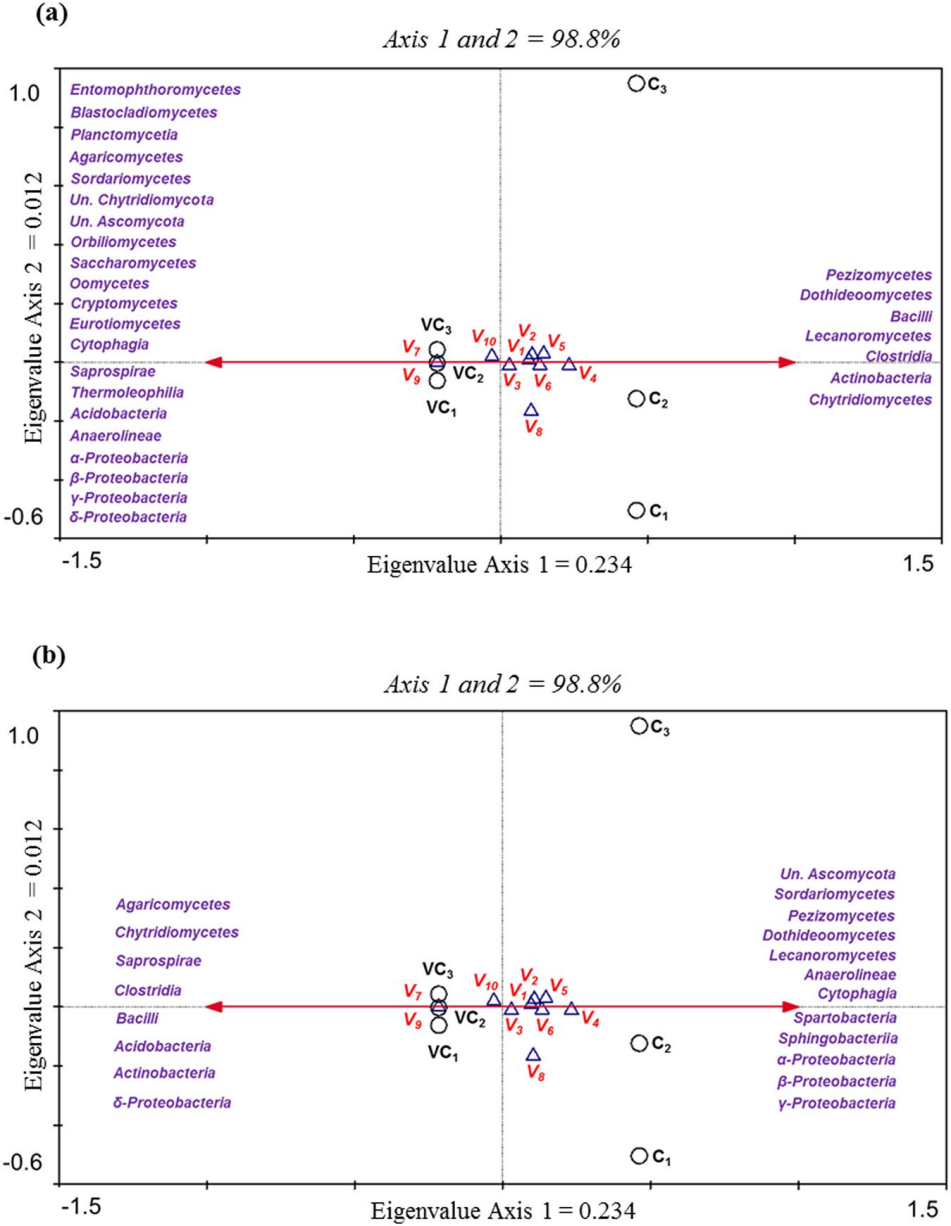
CCA triplot analysis of volatiles emitted by *Nerium oleander* plants grown in a potting soil initially containing a sterile (C: Control)/non sterile (VC: Vermicompost) inoculum and after attack by *Aphis nerii*. Arrows indicate the orientations of total (**a**) and active (**b**) rhizosphere microbiome. *V*_*1*_: 4-methoxy-benzaldehyde, *V*_*2*_: benzothiazole, *V*_*3*_: benzyl alcohol, *V*_*4*_: dichlorobenzoic acid, *V*_*5*_: alkylbenzenes, *V*_*6*_: 1,2,4-trimethylbenzene, *V*_*7*_: 2-decanone, *V*_*8*_: 2-undecanone, *V*_*9*_: 2-dodecanone, *V*_*10*_: tetradecane.

Analysis of similarity (ANOSIM) was used to statistically determine the effects of the initial inoculum on final rhizosphere microbial community structure after 3 months. This analysis generated an *R* value of 1, indicating total separation between soils at either the DNA (P = 0.098) or RNA (P = 0.094) level. Similarity percentage (SIMPER) analyses show the major peaks corresponding to the differences between the two treatments. With respect to total bacterial and fungal communities (Table 2), the OTUs assigned to 18 soil microbial classes accounted for 90% of total dissimilarity (Bray-Curtis dissimilarity = 26.5%). The members of classes *Deltaproteobacteria*, *Betaproteobacteria* and *Acidobacteria* accounted for roughly 25% of community variation between the two soils. With regard to active microbial communities (Bray-Curtis dissimilarity = 21.7%), 11 soil microbial classes accounted for 90% of total variation across different treatments, with *Pedosphaerae* (*Verrucomicrobiota*) and *Acidobacteria*, contributing to 25% of the dissimilarity between the two groups, as the most discriminant species (Table 3). To determine whether community composition affects volatile blend composition, canonical correspondence analysis (CCA) was performed. To test our hypothesis in this multitrophic system, volatiles were regarded as dependent analytical factors. CCA analysis carried out to examine the effect of total and active community composition on volatile emission resulted in a species-volatiles correlation of 0.999 and significant axes in both cases (trace = 0.234, F = 60.185, P = 0.0020), with CCA axis 1and axis 2 accounting for over 98% of variance. The results show two distinct groups corresponding to the two soils, each positively correlated with specific microbial classes at either DNA or RNA level (Fig. 6).

**Table 2:** SIMPER analysis identifies top abundant taxa that contributed most of the dissimilarities (>1%) between the total microbial rhizosphere communities from soil initially containing a sterile (Control)/non sterile (Vermicompost) inoculum.

Microbial class	average dissimilarity	% contribution to overall dissimilarity	% cumulative contribution to dissimilarity
*Deltaproteobacteria*	2.270	8.567	8.57
*Betaproteobacteria*	2.128	8.032	16.60
*Acidobacteria*	2.128	8.032	24.63
*Planctomycetia*	2.098	7.918	32.55
*Anaerolineae*	2.082	7.856	40.41
*Cytophagia*	2.065	7.792	48.20
*Thermoleophilia*	2.047	7.724	55.92
*Saprospirae*	2.028	7.653	63.57
*Cryptomycetes*	1.298	4.898	68.47
*Blastocladiomycetes*	1.280	4.832	73.31
*Orbiliomycetes*	0.667	2.515	75.82
*Saccharomycetes*	0.605	2.282	78.10
*Pezizomycetes*	0.599	2.260	80.36
*Sordariomycetes*	0.523	1.974	82.34
*Lecanoromycetes*	0.506	1.909	84.25
*Oomycetes*	0.485	1.829	86.08
*Chytridiomycetes*	0.444	1.674	87.75
*Unassigned Ascomycota*	0.402	1.516	89.27
*Clostridia*	0.399	1.507	90.77
*Alphaproteobacteria*	0.397	1.497	92.27
*Eurotiomycetes*	0.388	1.465	93.73
*Entomophthoromycetes*	0.354	1.334	95.07
*Gammaproteobacteria*	0.324	1.222	96.29
*Bacilli*	0.286	1.080	97.37
*Agaricomycetes*	0.247	0.934	98.30
*Unassigned Chytridiomycota*	0.202	0.764	99.07
*Dothideomycetes*	0.194	0.733	99.80
*Actinobacteria*	0.053	0.198	100.00

**Table 3:** SIMPER analysis identifies top abundant taxa that contributed most of the dissimilarities (>1%) between the active microbial rhizosphere communities from soil initially containing a sterile (Control)/non sterile (Vermicompost) inoculum.

Microbial class	average dissimilarity	% contribution to overall dissimilarity	% cumulative contribution to dissimilarity
*Pedosphaerae*	3.077	14.180	14.18
*Acidobacteria*	2.563	11.810	26.00
*Saprospirae*	2.483	11.440	37.44
*Anaerolineae*	2.422	11.170	48.61
*Sphingobacteriia*	2.380	10.970	59.58
*Dothideomycetes*	1.744	8.038	67.62
*Agaricomycetes*	1.718	7.919	75.54
*Pezizomycetes*	0.998	4.599	80.14
*Lecanoromycetes*	0.903	4.160	84.30
*Unassigned Ascomycota*	0.732	3.372	87.67
*Chytridiomycetes*	0.719	3.312	90.98
*Actinobacteria*	0.475	2.190	93.17
*Bacilli*	0.424	1.953	95.12
*Cytophagia*	0.351	1.618	96.74
*Sordariomycetes*	0.247	1.136	97.88
*Deltaproteobacteria*	0.148	0.682	98.56
*Clostridia*	0.078	0.359	98.92
*Spartobacteria*	0.077	0.356	99.28
*Gammaproteobacteria*	0.063	0.291	99.57
*Betaproteobacteria*	0.048	0.221	99.79
*Alphaproteobacteria*	0.046	0.212	100.00

## Discussion

This study is based on the “integration” approach which uses previous evidence concerning the role of the below-ground rhizosphere microbiome at above-ground third trophic level. We tested *N. oleander* plants grown in a potting soil initially containing a sterile/non-sterile olive waste vermicompost as inoculum. When attacked by *A. nerii* after 3 months of growth, SPME fiber analysis revealed the presence in both treatments of a characteristic blend of plant volatiles derived from two biosynthetic classes*:* fatty acid catabolites and aromatic-derived products. The composition of the aromatic blend was, in qualitative and quantitative terms, generally similar in both treatments, with comparable relative amounts of 4-methoxy-benzaldehyde, benzothiazole, benzyl alcohol, dichlorobenzoic acid, alkylbenzenes and 1,2,4-trimethylbenzene (Supplementary Fig. S5). Many volatile compounds containing an aromatic ring produced by the shikimate pathway have been described in a wide range of plant species regarded as HIPVs. For instance, 4-methoxy-benzaldehyde has been described in herbivore-infested *Arabidopsis thaliana* plants^19^; in aphid species, perception of this plant-specific volatile component assists olfactory discrimination between host and non-host plants^20^. The release of insect-induced benzothiazole in rice and sunflower plants damaged by *Tibraca limbativentris* and *Euschistus heros* has also been described^21^. Benzyl alcohol has been identified in *Camellia sinensis* and *Coffea canephora* under attack from different insect feeding guilds^22^ and even under mechanical damage conditions^23^. Dichlorobenzoic acid has been described as a functional analogue of the defence hormone salicylic acid, which acts as a bioactive plant defence-inducing compound^24^. Many different alkyl benzenes have also been retrieved from infested plants, especially in the presence of high densities of fungi^25^. Finally, for example, 1,2,4-trimethyl benzene has been characterized as a *Brassica oleracea* HIPV when infested with *Pieris rapae* larvae^26^. The volatile blends of fatty acid derivatives contained four aliphatic compounds: the methyl ketones 2-decanone, 2-undecanone and 2-dodecanone, as well as the alkane tetradecane, all low-molecular-weight substances previously classified as HIPVs. The results obtained by Lozano *et al*.^27^ indicate that 2-decanone is involved in attracting the parasitoids *Dendrosoter protuberans* and *Cheiropachus quadrum* to their host *Phloeotribus scarabaeoides*. Some studies have revealed the role played by 2-undecanone in an integrated pest management strategy for tomato^28^ and solanaceous crops, the latter due to its capacity to alter *Bactericera cockerelli* behavior^29^. However, other studies have also shown that 2-undecanone emissions do not differ between *Brassica rapa* plants exposed/not exposed by *Pieris brassicae* to leaf herbivory^30^. Hervibore-induced 2-dodecanone has been detected as a tomato volatile blend component^31^. The alkane tetradecane, a known semiochemical for many arthropods^32^, has also been identified as a volatile biomarker indicating damaged flower head tissue^33^. The most remarkable finding of our study was that three aliphatic compounds were mainly detected after an aphid attack in plants grown in the inoculated microbial soil. Neither 2-decanone nor 2-dodecanone was detected in any of the plants of the Control treatment, at least above the detection limit of the method and in the experimental conditions described above. The other key volatile, tetradecane, was emitted by all the infected plants, either grown in Control or Vermicompost conditions, being the emission significantly higher in the last.

The biosynthesis and regulation of plant volatiles have been widely studied^34^. Herbivore damage usually elicits phytohormone-mediated changes in the expression of genes involved in biosynthetic pathways for the production of plant volatiles. Variables influencing plant volatile emissions are multiple, including abiotic and biotic characteristics such as plant species, variety, phenology, physiology and nutritional quality, environmental conditions such as light, temperature and moisture status, herbivore density and population growth, as well as biotic and abiotic soil stresses^10^. In our experiment, carried out under controlled environmental conditions, any possible impact of these stresses was limited. Plants of the same variety were grown under similar environmental conditions and under the same physico-chemical soil characteristics and no differences in plant dry biomass were detected between the two treatments. Also, even though the pre-reproductive period of a mature *A. nerii* can be less than 2 days^35^, no visual differences in aphid population size among treatments were observed similarly to that noted by other authors in comparable experiments^36^, notwithstanding that aphid number was not actually quantified.

Under all the standardized conditions, the contrasting effect of the initial inocula on shifts in the emission of the fatty acid-derived volatile blends could be mainly attributed to the different microbial consortia developed in each treatment. In this context, the main cause of the synthesis of fatty acid-derived volatiles has been identified as the lipoxygenase pathway which is positively regulated by phytohormones such as jasmonic acid^37^. In general, although phloem feeders activate the salicylic acid-dependent shikimic acid pathway, the important role played by jasmonic acid in defence against aphids has also been demonstrated^38^. Globally, phytohormone crosstalk has been shown to be considerably involved in the biosynthesis of plant volatiles, although the mechanism by which the microbe interacts with the plant, which subsequently affects plant-insect interaction, has not been fully elucidated^39^. Beneficial micro-organisms such as mycorrizae and rhizobacteria have been demonstrated to play a role in modulating plant-induced systemic resistance, which is mediated by phytohormone signaling^40, 41^. However, studies focusing on the role of soil microorganisms in the modification of plant volatile emission are still scarce. Previous findings have shown that non-pathogenic root-associated microbes can have a positive or a negative effect on the attraction of third trophic level organisms through changes in the composition of the blend of herbivore-induced plant volatiles^14, 42^. Despite the necessity for further research in order to elucidate the mechanisms underlying these contrasting effects, the role assigned to microorganisms involves changes in defence-related signaling. Given that stress responses share signalling pathways regulated by defence-related phytohormones^43^, soil organisms could have the capacity to modulate the synthesis of fatty acid-derived volatiles. From a multitrophic perspective, the differences in the emission of selected aliphatic compounds by plants under aphid attack initiate a volatile blend attractive to a generalist predator. Thus, *C. carnea* adult females exhibit some degree of preference for certain volatile blends. It is well known that volatile plant secondary metabolites such as terpenes and aromatic compounds can be detected by the olfactory system of *C. carnea* adults to locate suitable hosts^44, 45^. Although these adults are not predatory, some research evidences mechanisms to attract females and concentrated them locally in the field to increase egg-laying intensity, to get advantages of the potential of *C. carnea* larvae as biological control agents^46^. It is possible to infer from our study that *C. carnea* adult females respond to a volatile blend, in which the presence of aliphatic compounds is a determining factor.

However, most studies focusing on below- and above-ground interactions involve single microbes, which differ greatly from natural soil conditions, highlight the importance of evaluating the entire soil microbiome when studying microbe-plant-insect interactions. Little research has been devoted to the impact of the soil microbiome on primary metabolite production in plants, which, in turn, determines insect feeding behavior^8^. It is still important to determine whether the soil microbiome modulates the biosynthesis of secondary metabolites involved in plant-insect interactions. In this study, we aimed to assess whether alterations in the microbial community composition of a sterile soil following vermicompost amendment modify generalist predator responses to shifts in volatile compound emission induced by a phytophagous attack. The effects of the vermicompost-borne microbial community on both the entire and functional rhizospere microbiome structure were evident after three months. Microbiome composition structurally evolved in line with the composition of the inoculums, while the chemical characteristics of the potting soil remained unchanged. Canonical Correspondence Analysis was used to determine whether community composition affects volatile blend composition. The results show two distinct groups corresponding to the two soils, each positively correlated with specific microbial classes at either the DNA or RNA level. It was then necessary to determine whether the plant-mediated effects of microbes on above-ground herbivores are dependent on microbial community composition and the role, if any, played by the microbial physiological state. The soil samples and volatiles matched different microbial classes depending on the total or active nature of the microbial population. As mentioned above, communication is well known to occur between plants and microorganisms, in which their signalling molecules play an important role. Active microorganisms may then be critically involved in this molecular dialogue, whose precise mechanisms in herbivore-induced plant volatile emission still need to be determined. In this study, Vermicompost treatment samples were clustered with the aliphatic volatiles 2-decanone, 2-dodecanone and tretadecane, and positively correlated with active fungi ascribed to classes *Chytridiomycetes* and *Agaricomycetes* and active bacteria of classes *Saprospirae*, *Deltaproteobacteria*, *Actinobacteria*, *Acidobacteria*, *Bacilli* and *Clostridia*. Previous studies of the relationship between microbiome members and plant volatile induction by herbivory have described the effects of soil bacteria ascribed to classes *Alpha-* and *Gamma-proteobacteria*, as well as endophytic fungi, but not those of the taxa mentioned above^42, 47, 48^, although it should be noted that these studies focus on the role of single species. However, from a metagenomics perspective, we did not find any remarkable differences in the structure of *Alpha-* and *Gamma-proteobacteria* bacterial communities, with virtually all the bacterial species ascribed to these classes sharing the soils of the two treatments (Supplementary Fig. S3).

Under our experimental conditions, we found some evidence to show the contribution of a group of functional rhizosphere bacteria and fungi to the diversity of plant volatile patterns. Given the absence of previous data on the role of free-living classes of fungi in indirect induced plant defence, we focused on a more in-depth analysis of the bacterial population. In this regard, we were unable to identify any differences in bacterial patterns between Vermicompost and Control rhizospheres, with both soils appearing to share virtually all bacterial species ascribed to the discriminant taxa, particularly at the RNA level (Supplementary Figs S2, S3). Although some authors have debated the key role played by rare species within the entire microbiome in plant-herbivore interactions^35^, we cannot attribute any critical function to species ascribed to those classes identified as discriminant between treatments. On the contrary, using a metagenomic approach, we showed that the principal difference mainly related to the relative abundance of functional microorganisms. In this regard, the estimators of alpha diversity^49^ indicated higher within-community diversity in the Vermicompost rhizosphere with respect to that found in the Control treatment. The *Chao 1* index^50^ and the Faith’s Phylogenetic Diversity^51^, based upon the number of rare OTUs and expressing the number of tree units found in a sample, respectively, confirmed that phylogenetic and functional structures of the bacterial communities differed between treatments. In addition, the beta diversity patterns, especially those including the phylogenetic distances between organisms, pointed to a medium-to-high degree of dissimilarity between the bacterial population structure in Vermicompost and Control rhizospheres.

## Concluding Remarks

In this study, the effects of below-ground microbes on indirect plant defences were evaluated. We aimed to determine whether alterations in the microbial community composition of a sterile soil following vermicompost amendment modify plant-insect interactions through shifts in volatile compound emission. We found that differences in the composition of the rhizosphere active microbiome correlate with those in the emission of selected aliphatic compounds by plants under aphid attack, which initiates a volatile blend attractive to a generalist predator. Although further research is required, our results suggest that functional interactions between soil microbes play a significant role in regulating the biosynthesis of volatile plant secondary metabolites. The functional soil microbiome is a factor which needs to be investigated in order to assess whether the plant-mediated effects of soil microorganisms on above-ground herbivores are species- or population structure-dependent.

## Materials and Methods

### Plants

The plant species used in the experiments was 3-month-old oleander (*Nerium oleander* L. Apocynaceae) grown in a greenhouse nursery (25–30 °C, 60–80% RH, 16:8 h L:D). The roots were washed by being dipped in sterile water to remove soil and were then individually transplanted to a pot filled with the potting soil mixture. At harvest (90 days after planting), the plants were removed and separated into shoots and roots. The shoots were oven-dried at 60 °C for 48 h and their mass determined on an analytical balance.

### Potting soil

A mixture of sand and loamy clay soil (1:1 v:v), previously sterilized by fractional sterilization (tyndallization; 100 °C, 60 min, 3 days), was used as potting medium. The soil was a calcareous loam (Typic Xerorthent)^52^ collected from an agricultural field (0–20 cm in depth) in Granada, Spain. Tyndallization involves killing vegetative cells and some spores at the initial heating stage; additional heat resistant spores germinate and are killed at a later heating stage. This low temperature sterilization technique preserves soil structure and quality more effectively than autoclaving at 121 °C^53^. The tyndallized soil characteristics were as follows: 0.9 g kg^−1^ SOC (soil organic carbon), 1.6 g kg^−1^ total N, pH (H_2_O) 7.5.

We used a vermicompost from olive-mill waste produced at the EEZ-CSIC facility (Granada, Spain), as described in Vivas *et al*.^54^, as microbial inoculum. In order to attain a soil organic carbon content of 30 g kg^−1^, 1,000 g of the soil mixture was placed in 2-l black pots and thoroughly mixed with the vermicompost at a rate corresponding to 50 g kg^−1^ (Vermicompost treatment). Soil amended with the same amount of vermicompost sterilized by tyndallization was used as control (Control treatment). Soil moisture content was adjusted to approximately 60% and maintained at this level by irrigation with sterilized deionized water during the experiment. Three replicates per treatment were arranged in randomized blocks in the greenhouse (25 °C, 60–80% RH, 16:8 h L:D).

### Predators

The *Chrysoperla carnea* Steph. (Neuroptera: Chrysopidae) larvae were supplied by Koppert Biological Systems (La Mojonera, Almería, Spain). Larvae were individually reared in Petri dishes and fed on *Ephestia kuehniella* Zell. (Lepidoptera: Pyralidae) eggs. Upon emergence, *C. carnea* adults were collected daily and kept in boxes (28 cm diameter, 15 cm high) with an ovipositing surface; they were then fed on honey:pollen (1:1, v-v) and mineral water and maintained in a controlled environment cabinet at 25 °C, 50–60% RH and 16:8 h L:D for 2–3 days. Adult *C. carnea* were sexed by examining the ventral abdominal tip surface. Only females were used for bioassays.

### Phytophagous insects

*Aphis nerii* Boy. (Homoptera: Aphididae) adults were taken from 20-y-old *N. oleander* plants located in Gójar, Granada, Spain. 20 individuals were reared on 1-y-old oleander plants maintained in a chamber at 25 °C, 50–60% RH and 16:8 h L:D. The plants were covered with fine mesh netting to prevent *A. nerii* emigration. When the plants were badly damaged by aphids, they were replaced by fresh plants after aphid migration to the healthy plants. Approximately 7 generations of aphids were produced before being used for the experiments.

### Experimental design

A closed-system Y-tube olfactometer (ID 3 cm; stem 10 cm, arms 8 cm; stem-arm angle 130°) was used to assess choice of predator *C. carnea* between the two treatments after a 3-month *N. oleander* growing period (Fig. 1). Two glass chambers (40 × 40 × 140 cm), sufficiently large to accommodate aboveground plant tissues were connected from the top to the Y-shaped glass tubing of the olfactometer by transparent polytetrafluoroethylene. An SPME fiber was inserted into each arm of the olfactometer in order to collect volatiles. Using air pressure, synthetic pure air, at an airflow rate of 1.2 l min^−1^ per channel^55^, was drawn in though the bottom of the chambers.

Firstly, uninfested plants were tested in the chambers connected to the olfactometer, and the volatiles were recovered. To infest *N. oleander*, 20 wingless *A. nerii* adults were introduced at the top of the plant. After inoculation, the plants were re-introduced into the glass chambers. The aphids were allowed to feed for 48 h, and tests were conducted three days after inoculation.

Behavioural tests of predator *C. carnea* were carried out under artificial light between 09:00 and 18:00 h at 28 ± 2 °C. A white circular paperboard arena was placed around the olfactometer to prevent visual disturbances. Adult *C. carnea* females were inserted into the single branch of the olfactometer and were left to choose between the two branches of the device, with a maximum observation period of 5 min. If the insects, which were used only once and then discarded, did not attain a length of at least 4 cm along the arm connected to the test chambers, they were excluded from the data analysis. To rule out directional bias, the olfactometer was washed in hot water, rinsed in sterilized deionized water and dried in an oven at 60 °C before each experiment. The Y-tube was also rotated 180° after each test. The position of the chambers was changed after every two tests. The pairwise experiment on the behaviour of two plants was repeated three times every other day. In each assay, approximately 7 *C. carnea* females were used. A total of 22 behavioural assays were carried out with *C. carnea* females from three different rearing groups (blocks).

### Volatiles

The volatiles emitted by *N. oleander* were sampled using SPME on day 3 for 9 h from 09:00 to 18:00 h. The SPME fibers (50/30 μm DVB/CAR/PDMS Stableflex 23Ga, Autosampler, 3pk, SUPELCO, Bellefonte, PA, USA) were preconditioned prior to analysis at 250 °C for 30 min. After the equilibration period, the fibers were exposed to the headspace of each Y-tube olfactometer arm. After completion of sampling, the fiber was withdrawn into the needle and inserted into the GC–MS system injection port in splitless injection mode at an injector temperature of 250 °C.

Gas chromatography (GC) analyses were conducted on a Varian 450-GC gas chromatograph fitted with a 1079 injector in split/splitless mode, a CTC Analytics CombiPal refrigerated autosampler and a Varian 240 Ion Trap as a mass spectrometer detector. A FactorFour VF-5ms (30 m × 0.25 mm × 0.25 μm) fused silica capillary column was also used.

The initial gas chromatography oven temperature was 50 °C for 5 min which was then increased to 260 °C at 10 °C min^−1^. It was then raised to a temperature of 300 °C at 30 °C min^−1^, which was maintained for 1 min with an injection of 1 μl (300 °C) in splitless mode (1 min). The carrier gas was He at 1 ml min^−1^. Electron impact ionization and detection in full scan (*m*/*z* 40 to 450) modes were carried out. The transfer line and trap temperatures were 290 and 210 °C, respectively. Peaks were identified by comparing the volatile sample mass spectra with spectra in the NIST08 Mass Spectral Database (MS Workstation 6.9.1. software). When necessary, the retention index (RI) was calculated for each volatile compound using the retention times of a homologous series of n-alkanes and by comparing the RI with that of compounds analyzed under similar conditions in the literature to confirm the identity of volatile compounds.

### Rhizosphere soil samples

Rhizosphere soil was collected in two steps. First, the root system was separated from the bulk soil by gentle shaking, and the remaining soil was then removed from the roots by more vigorous shaking. Soil still adhering to the roots was removed using a sterile dissecting probe and collected for use as rhizosphere soil. Root-associated soil samples from each pot were placed in separate polyethylene bags and immediately stored at −80 °C for subsequent molecular analyses.

### 16S/18S rRNA gene sequence analysis

For each rhizosphere soil sample replicate, total DNA was separately extracted from four 1 g subsamples using the bead-beating method following the manufacturer’s instructions for the MoBio UltraClean Soil DNA Isolation kit (MoBio Laboratories, Solana Beach, CA, USA). The extracts were pooled and further concentrated at 35 °C to a final volume of 20 μl, with the aid of a Savant Speedvac® concentrator. Total RNA was extracted from four 2 g subsamples of each replicate according to the manufacturer’s instructions for the MoBio RNA PowerSoil Total RNA Isolation kit (MoBio laboratories, Solana Beach, CA, USA). To remove residual DNA, the DNase I enzyme was added using the Roche RNase-Free DNase set (Roche Applied Science, Penzberg, Germany) according to the manufacturer’s instructions. The extracts were pooled and further concentrated at 35 °C to a final volume of 80 μl with the aid of a Savant Speedvac® concentrator. The cDNA was synthesized from 1–2 μg of total RNA-DNase using the Transcriptor High Fidelity cDNA Synthesis Kit according to the manufacturer’s instructions (Roche Applied Science, Penzberg, Germany). The synthesis reaction was carried out at 50 °C for 30 min. The concentration and quality of the final DNA/RNA/cDNA samples were checked by a Nanodrop® ND-100 spectrometer (Nanodrop Technologies, Wilmington, DE, USA).

The metagenome and transcriptome libraries of the bacterial 16S rRNA gene were generated in pooled soil samples using the primers S-D-Bact-0341-b-S-17/S-D-Bact-0785-a-A-21 reported by Klindworth *et al*.^56^ (non-underlined sequences) and were fused with underlined Illumina adapter overhang nucleotide sequences. To amplify V3-V4 hypervariable regions of the 16S rRNA gene, the following primer sequences were used: 5′-TCGTCGGCAGCGTCAGATGTGTATAAGAGACAGCCTACGGGNGGCWGCAG-3′ and 5′ GTCTCGTGGGCTCGGAGATGTGTATAAGAGACAGGACTACHVGGGTATCTAATCC-3′. The amplified region was approximately 464 bp. For each library, triplicate soil PCR products with unique indexes were mixed in equal nanogram quantities and sequenced on the Illumina MiSeq platform using a 2 × 250 nucleotide paired-end protocol (Era7 Bioinformatics, Granada, Spain).

Metagenomic and transcriptomic analyses of the fungal 18S rRNA gene were performed on the degenerate primers 563 f (5′-GCCAGCAVCYGCGGTAAY-3′) and 1132r (5′-CCGTCAATTHCTTYAART-3′) designed by Hugerth *et al*.^57^. To prepare libraries for Illumina sequencing, primers 563 f and 1132r were fused with the Illumina adapter overhang nucleotide sequences. The primers were used to amplify the V4 region of the 18S rRNA gene, with the amplicon expected to measure approximately 569 bp. For each library, triplicate soil PCR products with unique indexes were mixed in equal nanogram quantities and sequenced on the Illumina MiSeq platform using a 2 × 300 nucleotide paired-end protocol (Era7 Bioinformatics, Granada, Spain).

The resulting sequences from 16S and 18S rRNA gene libraries were assessed and filtered according to base quality using the FASQC tool (http://www.bioinformatics.babraham.ac.uk/projects/fastqc/). The quality-filtered Illumina paired-end reads were merged using FLASH^58^. The non-merged reads considered to be invalid for analysis were discarded. The successfully merged fragments were assigned to a taxonomic tree node based on sequence similarity to 16S and 18S rRNA genes extracted from the RNA central database (http://rnacentral.org/) which includes rRNAs from a wide range of major databases such as SILVA, GreenGenes, RDP, RefSeq and ENA. The NCBI taxonomy was used; for taxonomic assignment, we used the MG7 method^59^ which is based on exhaustive BLAST comparison of each read, covering almost the whole length of the sequence, against the 16S and 18S ribosomal RNA database. This method discarded chimeric sequences as no-hit reads, given that the reference database only contains 16S and 18S sequences and chimeric sequences have a BLAST no-hit result. In addition to chimeric sequences, the majority of no-hits detected in all the samples correspond to phiX reads that are used as controls in the Illumina library preparation protocol in the sequencing experiment. The bacteria detected in the samples were identified on the basis of the BLAST results. Taxonomic assignment was carried out using the BBH (best BLAST hit) and LCA (Lowest Common Ancestor) assignment paradigms. Taxonomic profiling was studied at the sample level^60^. An additional filtering step was carried out exclusively for the 18S rRNA dataset, with only reads belonging to the Kingdom Fungi being kept for downstream analyses.

All original Illumina sequence data were deposited in the Sequence Read Archive (SRA) service of the European Bioinformatics Institute (EBI) database (BioProject ID: PRJNA313153, accession numbers SRX1795432, SRX1795541, SRX1798892, SRX1798901, SRX1795397, SRX1795517, SRX1798890, SRX1798895).

### Chemical analyses

Air-dried rhizosphere soil samples were used to determinate chemical properties. Total N and SOC were determined with the aid of the Leco-TruSpec CN elemental analyzer (LECO Corp., St Joseph, MI, USA). Total mineral content was determined by the digestion method with HNO_3_ 65%:HCl 35% (1:3; v-v) followed by analysis using inductively coupled plasma optical emission spectrometry (ICP-OES) (ICP 720-ES, Agilent, Santa Clara, USA).

### Data analysis

The results of the chemical and volatile analyses were the means of 3 replicates. The data were subjected to factorial analysis of variance (ANOVA) using PAST (Paleontological Statistics) software program v3.14^61^.

Data on the behaviour of *C. carnea* were analysed to account for differences between treatments by using generalized linear mixed models (GLMMs). A Poisson error structure and log-link function were used to build these models, with the response variable being the count of *C. carnea* females located at the end of each Y-tube olfactometer branch^62^. We generated a set of models composed of different combinations of the fixed “treatment” and “block” factors and the random “assay” factor. By their nature, blocks should be regarded as a random factor; however, because this variable contains only three levels, a fixed factor is recommended^63^. We also tested a set of models with the block variable treated as a random factor and obtained the same results. The most complex of the eight plausible models we constructed, containing all possible combinations of the variables mentioned above, was the following:

$$C.\,carnea\,individuals=\alpha +{\beta }_{1}\,treatment+{\beta }_{2}\,block+{\varepsilon }_{assay}$$

where $\alpha$ represents the intercept of the model; ${\beta }_{1}$ is the estimated value of the treatment effect; ${\beta }_{2}$ is the estimated value of the block effect; and ${\varepsilon }_{{assay}}$ is the estimated error associated with the assays carried out. Alternative models were compared using the Akaike Information Criterion (AIC_c_) corrected for small sample size^64^. Models showing a difference in AIC_c_ > 2 indicate that the worst model has virtually no support and can be ruled out. The selected model was tested to account for unsuited error structure approach with DHARMa package ver. 0.1.3 written for the R environment^65^, given that the error structure chosen was appropriate for this type of analysis.

An additional analysis of bacterial sequences was carried out using QIIME v1.9.1^66^. The raw files from Illumina paired-end sequencing (R1 and R2) were merged. Quality filtering was then performed using Phred^67, 68^, with a Phred quality score of Q20. The FASTA files obtained were brought together in a single file. With the aid of UCLUST^69^, an OTU clustering procedure was performed with a 97% similarity threshold. To facilitate further analysis, a representative set of sequences was selected. An OTU table in biom format^70^ was obtained to further analyse alfa and beta diversity using different metrics. For the phylogenetic analysis of the representative set of sequences, an alignment using PyNAST^71^ was carried out, the highly variable regions of sequences were removed and, finally, the phylogenetic tree was obtained with the aid of FastTree^72^. The phylogenetic tree in newick format is necessary to calculate alfa diversity using the PD_whole_tree metric (Faith’s Phylogenetic Diversity) and beta diversity using the unweighted_unifrac and weighted_unifrac methods.

Analysis of similarity (ANOSIM) and similarity percentage (SIMPER) analyses were performed on total and active microbial communities (OTUs, 16S and 18S ribosomal amplicon pyrosequencing) using PAST software v3.14. Distance indices were calculated with the aid of the Bray-Curtis method. Statistical significance was computed by permutation of group membership with 9,999 replicates. ANOSIM generated an *R* value, whose magnitude indicates the degree of separation between groups, with a score of 1 indicating total separation and 0 no separation.

The relationship between plant volatile blends and rhizosphere microbiome composition (OTUs, 16S and 18S ribosomal amplicon pyrosequencing) was determined by canonical correspondence analysis in CANOCO 4.5^73^. OTU patterns were fitted to volatile blends by using the Monte Carlo permutation test (499 permutations) at a 95% confidence level. Community similarities were graphed by using an ordination triplot technique with scaling focused on inter-sample differences.

## Electronic supplementary material

Supplementary Info
