# Supplementary material for: Bottom-up effects on herbivore-induced plant defences: a case study based on compositional patterns of rhizosphere microbial communities
Source: Sci Rep. 2017 Jul 24;7:6251. doi: 10.1038/s41598-017-06714-x (PMC5524984; doi:10.1038/s41598-017-06714-x)

Bottom-up effects on herbivore-induced plant defences: a case study based on compositional patterns of rhizosphere microbial communities

Emilio Benítez, Daniel Paredes, Estefanía Rodríguez, Diana Aldana, Mónica González, Rogelio Nogales, Mercedes Campos, Beatriz Moreno

Supplementary Table S1. Chemical parameters of potting soil initially containing a sterile (Control)/non sterile (Vermicompost) inoculum.

	Control	Vermicompost
Al (mg kg^{-1})	11158	11440
As (mg kg^{-1})	1.40	1.31
Ca (g kg^{-1})	89.48	89.28
Cd (mg kg^{-1})	n.d.	n.d.
Co (mg kg^{-1})	n.d.	n.d.
Cr (mg kg^{-1})	19.02	20.06
Cu (mg kg^{-1})	16.76	19.16
Fe (mg kg^{-1})	6361	6558
Hg (mg kg^{-1})	n.d.	n.d.
K (g kg^{-1})	3.84	4.12
Li (mg kg^{-1})	7.78	8.07
Mg (mg kg^{-1})	6538	6636
Mn (mg kg^{-1})	238	237
Mo (mg kg^{-1})	n.d.	n.d.
Na (mg kg^{-1})	320	393
Ni (mg kg^{-1})	14.42	13.93
P (g kg^{-1})	1.19	1.28
Pb (mg kg^{-1})	6.99	5.46
S (mg kg^{-1})	659	882
Se (mg kg^{-1})	n.d.	n.d.
Si (mg kg^{-1})	3390	4161
Ti (mg kg^{-1})	578	618
V (mg kg^{-1})	16.50	17.57
Zn(mg kg^{-1})	29.80	33.37
N (g kg^{-1})	2.4	2.6
SOC (g kg^{-1})	25.7	28.3
C:N	10.7	10.9

n.d: not detected

Supplementary Fig. S1. Krona chart of the total bacteria represented by 16S rRNA gene sequences recovered from soil initially containing a sterile (Control)/non sterile (Vermicompost) inoculum.

Control

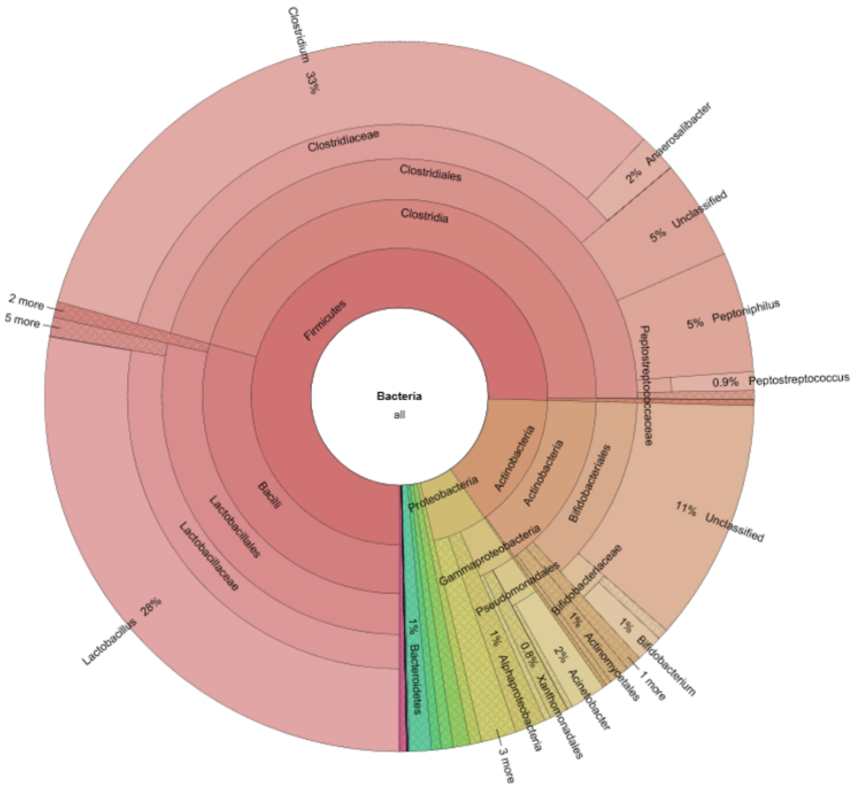

Vermicompost

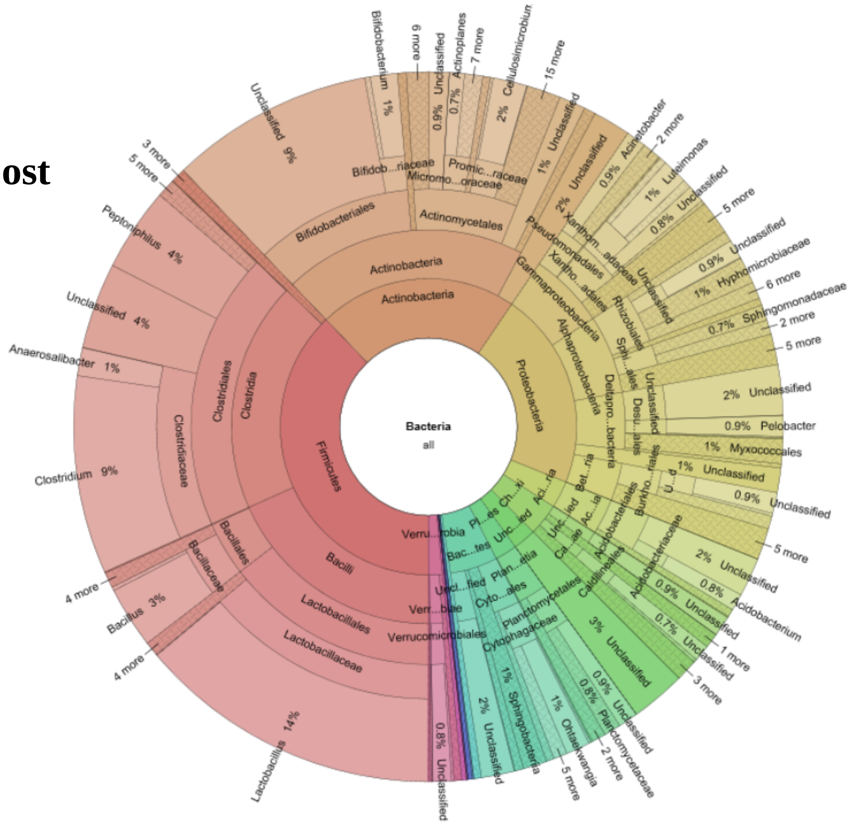

Supplementary Fig. S2. Krona chart of the active bacteria represented by 16S rRNA transcripts sequences recovered from soil initially containing a sterile (Control)/non sterile (Vermicompost) inoculum.

Control

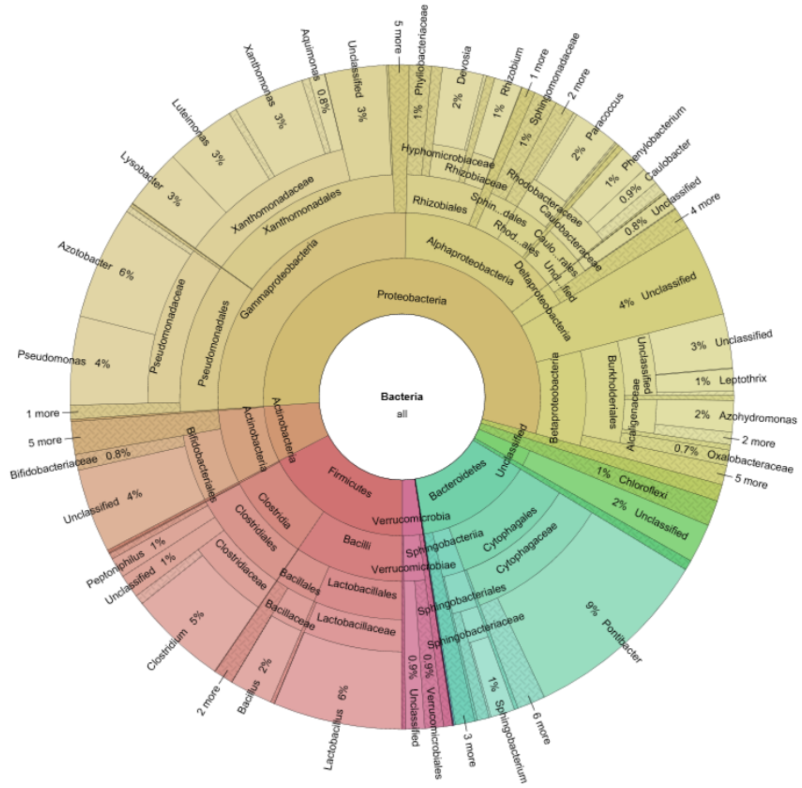

Vermicompost

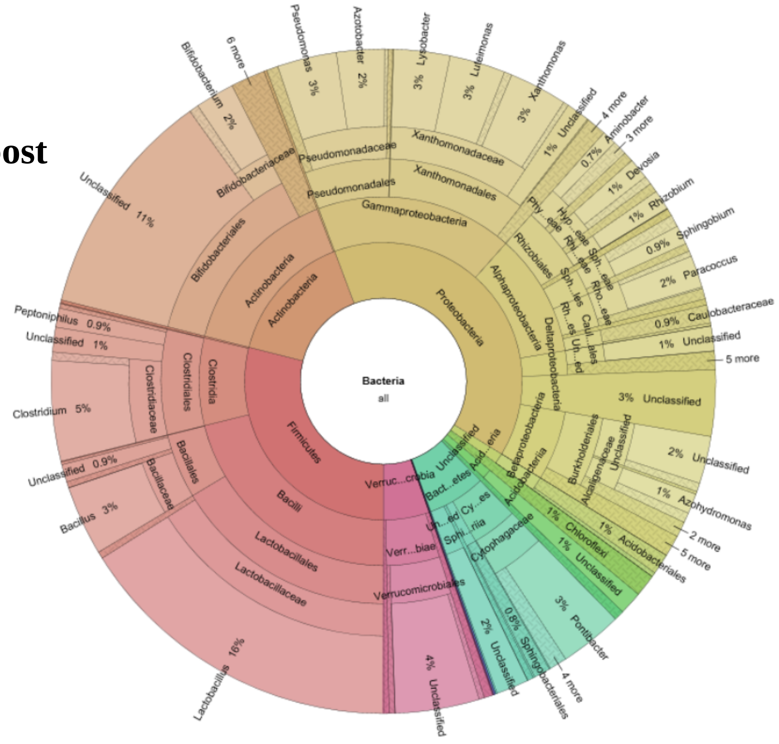

Supplementary Fig. S3. Krona chart of the active bacteria (selected groups) represented by 16S rRNA transcripts sequences recovered from soil initially containing a sterile (Control)/non sterile (Vermicompost) inoculum.

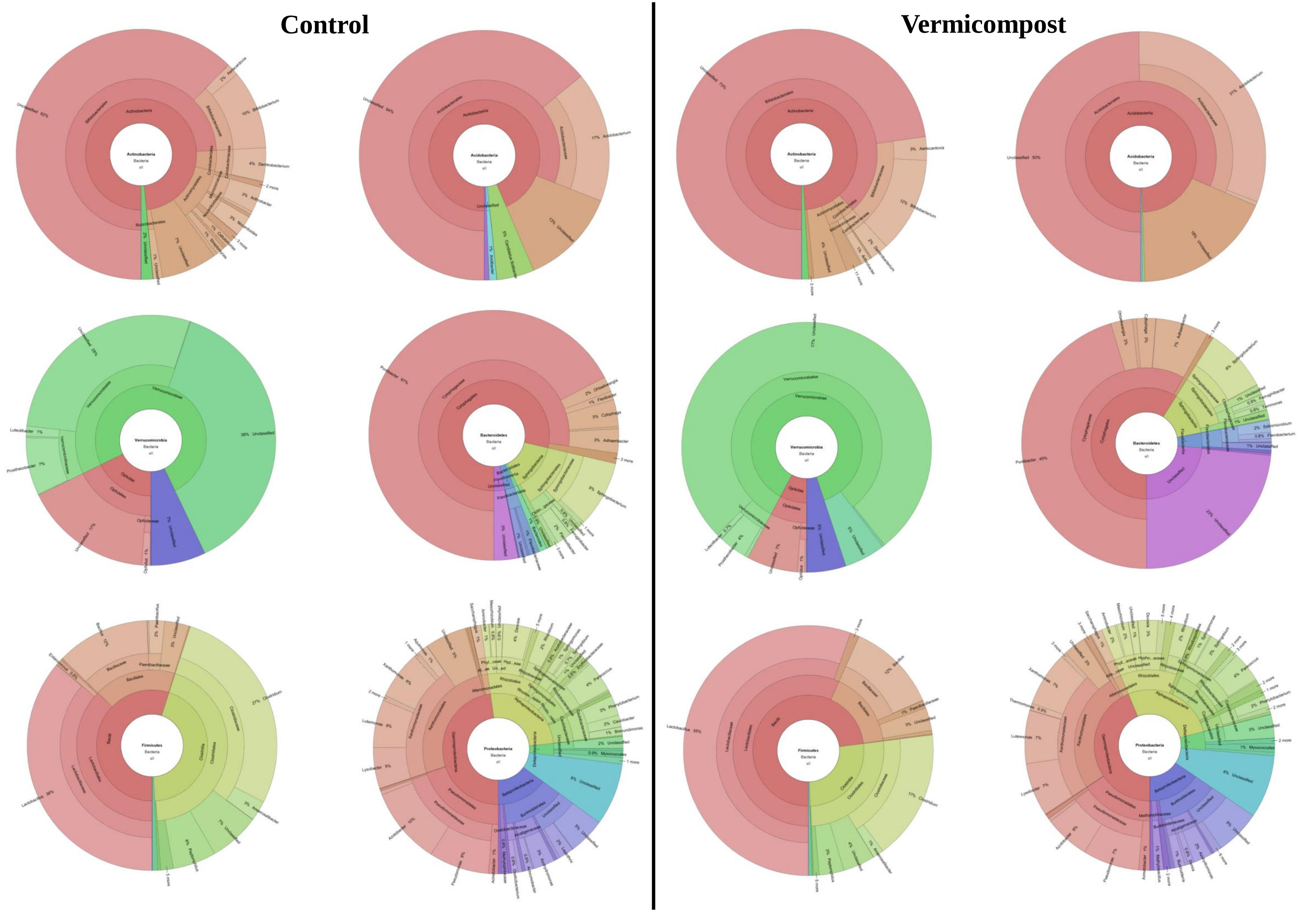

Control **Vermicompost**

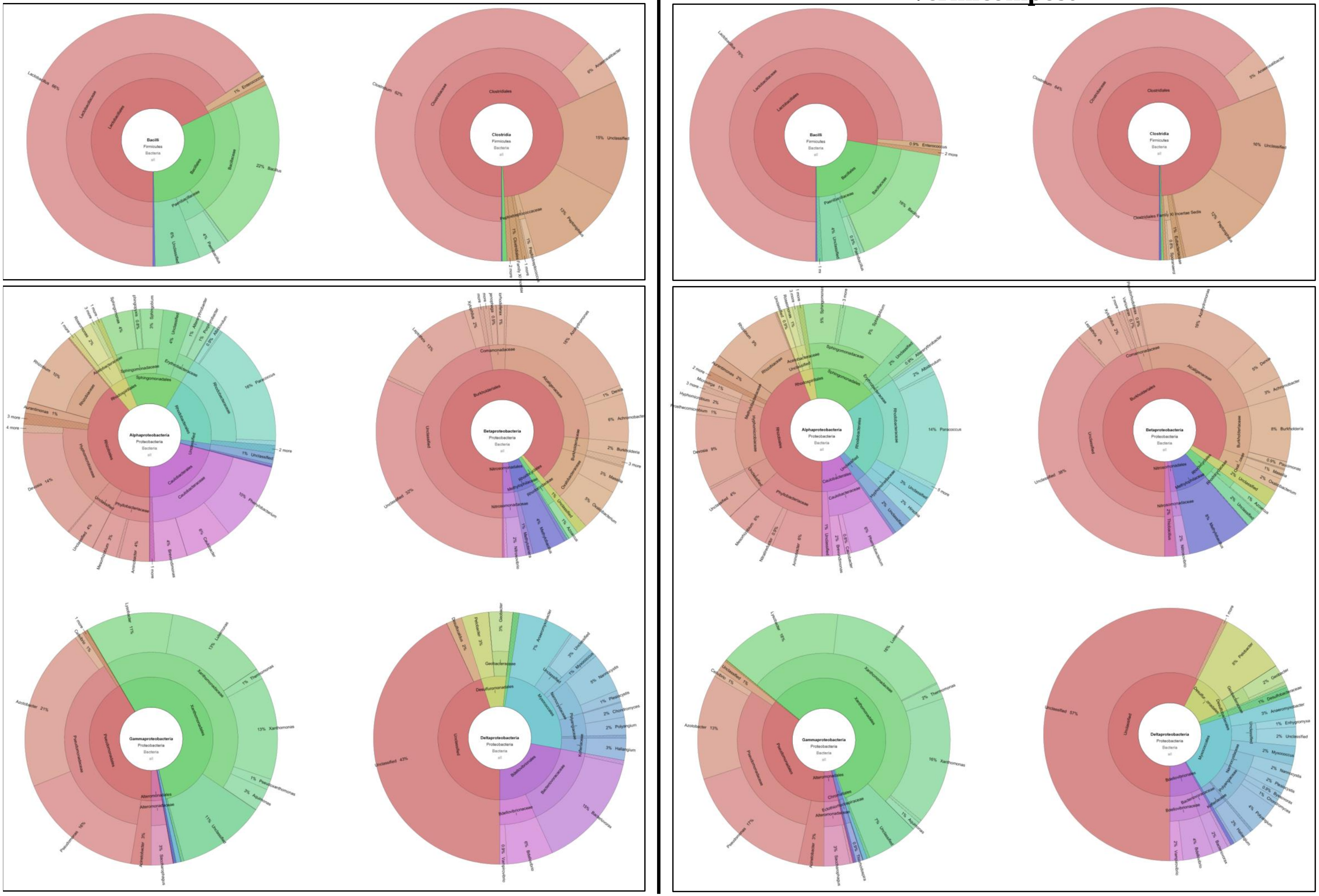

Supplementary Fig. S4. Rarefaction curves and alpha diversity values of 16S rRNA sequences recovered from soil initially containing a sterile (Control)/non sterile (Vermicompost) inoculum at both DNA and RNA levels.

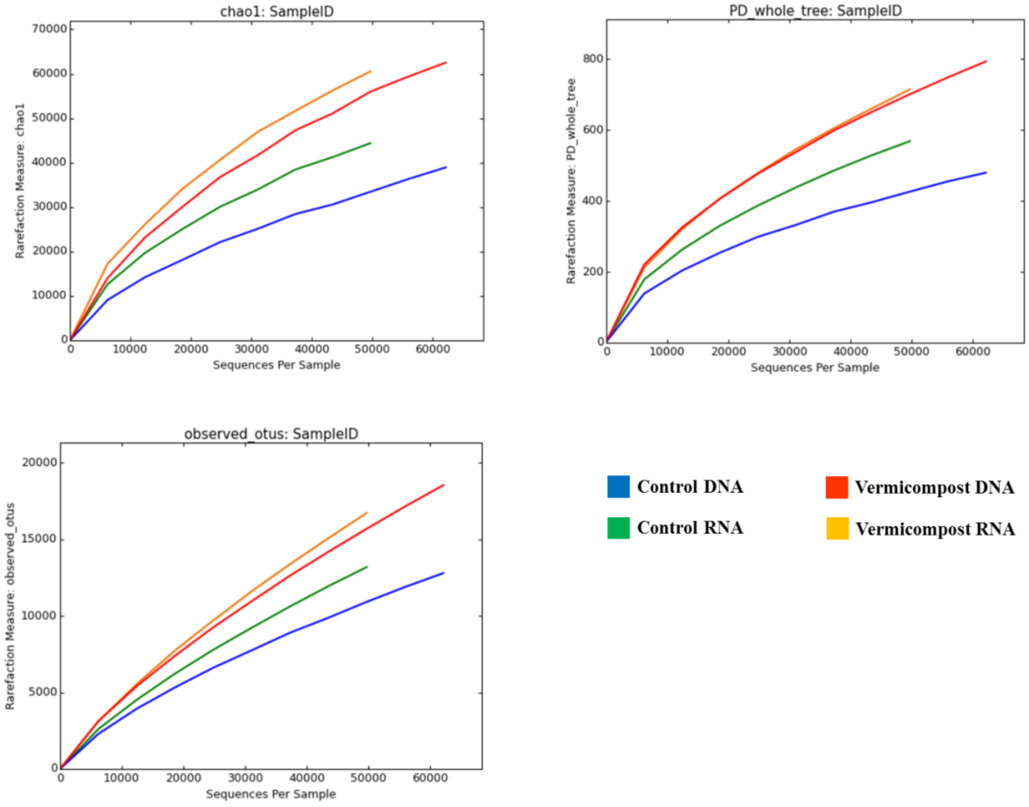

	Chao1	PD whole tree (Faith's Phylogenetic Diversity)	Observed OTUs
Control DNA	39.366	444	13.037
Vermicompost DNA	56.493	642	18.385
Control RNA	52.217	593	16.140
Vermicompost RNA	64.404	766	26.392

Supplementary Fig. 5. Volatile blends emitted by *Nerium oleander* plants grown in a potting soil initially containing a sterile (Control)/non sterile (Vermicompost) inoculum and after attack by *Aphis nerii*.

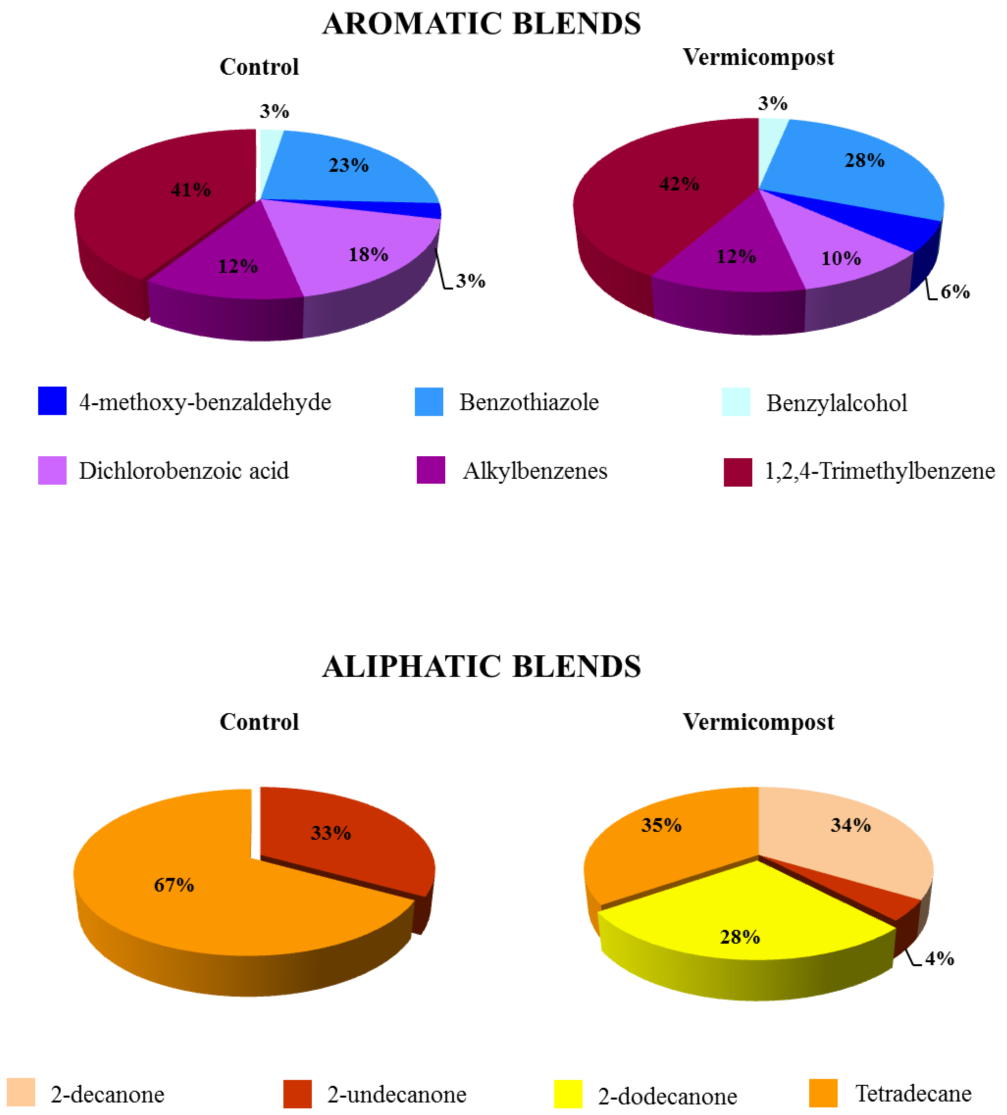

Supplement: Supplementary file 1 — Supplementary Info [file 41598_2017_6714_MOESM1_ESM.pdf]
